# Meetings of Societies

**Published:** 1899-12

**Authors:** 


### MEETINGS OF SOCIETIES.

### Bristol Medico-Chirurgical Society.

*Annual Meeting, October 11th, 1899.*

Dr. R. Roxburgh, President, in the Chair.

The minutes of the last meeting having been read and confirmed, the President resigned the chair in favour of the President-elect, Mr. W. H. Harsant.

Mr. Nelson C. Dobson moved, and Dr. J. E. Shaw seconded, a vote of thanks to the retiring president for the admirable manner in which he had conducted the presidential duties during his year of office. The resolution having been carried with acclamation, Dr. Roxburgh replied.

The President then read his address, which will be found on pages 297—314, and at its conclusion Dr. A. J. Harrison proposed, and Dr. Shingleton Smith seconded, a vote of thanks to Mr. Harsant for his interesting address. This also was carried with acclamation.

The Hon. Secretary (Mr. Paul Bush) read his Annual Report, which showed that there was a credit balance on the ordinary account of £85 : 19 : 6, as against £47 : 3 : 5 at the close of the previous session; and a balance in hand on the *Journal* account of £118 : 17 : 8, as against £103 : 9 : 4 for the year 1897. The large balance on the ordinary account was due to the receipt of £184 : 16 : 0 received for subscriptions, this sum being the largest amount ever received in one year.—The President moved, "That the report of the Hon. Secretary be adopted, and that he be thanked for his services." This was seconded by Mr. C. E. S. Flemming, and carried.

Dr. B. M. H. Rogers, late Editorial Secretary of the *Journal*, read his report, which stated that in 1898 £224 : 16 : 11 had been received and £206 : 17 : 10 spent on the *Journal*, leaving a credit balance of £17 : 19 : 1.—Mr. A. W. Prichard proposed, "That the report be adopted, and that Dr. Rogers be thanked for his services." This was seconded by Mr. S. H. Swayne, and carried.

Mr. L. M. Griffiths, on behalf of the Committee of the Bristol Medical Library, read a report which showed that, including 798 duplicates, the library of the Society contained 8,177 volumes. During the year 1,088 volumes were added, and 91 given to other libraries. The Society is in regular receipt of 188 periodicals. The names of donors and the attendances of the *Journal* Committee were read. The Bristol Medical Library, to which the members of the Society have access, contains 19,982 volumes and 224 current periodicals.—It was proposed by Mr. J. Dacre, and seconded by Mr. A. N. Godby Gibbs, "That the report be adopted, and that Mr. Griffiths be thanked for his services." This was carried.

Dr. Roxburgh proposed, and Mr. Munro Smith seconded, Dr. D. S. Davies as President-elect. This was carried.

Dr. R. Lansdown proposed, and Dr. P. Watson Williams seconded, the re-election of Mr. Bush as Hon. Secretary. This was carried.

Dr. Shingleton Smith proposed, and Mr. L. M. Griffiths seconded, that the six elected Members of the Committee be Dr. J. Michell Clarke, Mr. J. Dacre, Dr. B. M. H. Rogers, Mr. G. Munro Smith, Mr. James Taylor, and Dr. H. Waldo. This was carried.

Mr. Bush proposed, and Dr. Harrison seconded, that the representatives of the Society on the Committee of the Bristol Medical Library consist of Mr. L. M. Griffiths, Mr. G. Munro Smith, and Mr. J. Taylor. This was carried.

---

*November 8th, 1899.*

Mr. W. H. Harsant, President, in the Chair.

The minutes of the last meeting were read and confirmed. Mr. L. W. Powell, Dr. A. S. Wohlmann, and Dr. J. Young were elected members of the Society.

Dr. W. H. C. Newnham showed a specimen of ruptured tubal pregnancy from a woman aged 28. After taking some ecbolic medicine, procured from a chemist, vaginal hemorrhage commenced, and lasted for six days before the woman was brought to the General Hospital. The abdomen was opened and found full of bloodclot. Slight peritonitis was present. The operation was completed, but the patient never properly recovered from it, and died on the third day.—Dr. Aust Lawrence drew attention to the necessity of early diagnosis in these cases, and illustrated his remarks with certain cases. He asked Dr. Newnham's opinion on the drainage of the abdominal cavity, his own being against it if the case was seen early. He had known septic peritonitis to follow drainage, so that in future he would, after removal of the clot and such cleansing of the peritoneum as the patient's condition would allow, pour in saline fluid and suture up completely.—Mr. Paul Bush advocated rapid removal of the clot, as little manipulation and irrigation of the intestines as possible, and complete closure.—Dr. W. C. Swayne enquired if the abortion was the result of the medicine, as such drugs were, as a rule, only drastic purgatives. He advocated the use of normal saline fluid being left in the abdominal cavity.—The President referred to the necessity of early diagnosis, but admitted that in many cases this was very difficult. —Dr. Newnham, in reply, said that in this case the symptoms were not so severe as they usually are in ruptured tubal pregnancies, and his belief was that the escape of blood was slow. He questioned whether blood could remain six days in the abdominal cavity without becoming infected from the intestines. He used saline irrigation, and did not drain, and in his opinion the drug was not the cause of the abortion.

Dr. T. Fisher read a paper on œdema of the eyelids, with intermittent albuminuria, in children. He had recently seen some debilitated children in whose urine he found traces of albumin at times. In some of them he obtained a history of œdematous eyelids, but in others was not able to do so. Transient albuminuria is found in dyspepsia and in other conditions in children, but it does not seem to affect their health in some cases.—Dr. H. Waldo asked Dr. Fisher's view of the prognosis in these cases.—Dr. B. M. H. Rogers said swelling of the eyelids was by no means an uncommon thing with children quite apart from albuminuria, and that albumin was often present at times when there was no œdema or swelling of the eyelids.—Dr. F. H. Edgeworth asked if there were any signs of arterial degeneration in these cases, any casts or albumose in the urine.—Dr. G. Parker said many cases of albuminuria were due to the circulation of toxins in the blood of children; the same conditions did not occur in adults. He referred to Henoch's purpura and Quincke's œdema, and other causes of œdema in children.—Dr. Markham Skerritt said he had seen albumose precede serum-albumin, and chronic renal disease develop subsequently. Had Dr. Fisher found albumose without serum-albumin?—Dr. Michell Clarke did not think these cases very uncommon, particularly when the twelve o'clock urine was examined. A saline caused disappearance of the albumin. He doubted the existence of "physiological albuminuria," and considered examination of the vascular system very important.—Mr. C. H. Walker said he had often been told in the eye department that swelling of the eyelids was present in children in the morning, but he thought it was often a maternal imagination. He found no changes in the fundus in these cases.—Dr. Shingleton Smith thought intermittent albuminuria was a disease, and he did not accept the theory of physiological albuminuria.—Dr. Fisher, in reply, said he did not only rely on parents' statements, as he had seen the œdema himself. He had not tested for albumose or examined for casts, and in only one case had he found vascular changes. He did not believe crying produced œdema of the lids, though it did swelling.

Dr. Michell Clarke read the notes of two sporadic cases of cerebro-spinal fever, aged 10 and 19 respectively. In the former the illness lasted eight days; in the latter only forty-eight hours, and in this case was ushered in by a general epileptiform convulsion. There were herpes labialis, high and irregular fever, delirium, and the usual symptoms of cerebro-spinal fever in each; in addition, a marked feature of the illness was the pronounced restlessness. Death was preceded by coma. Necropsy in each case showed the presence of a bright yellow exudation over the pia mater, especially over the convexity of the cerebral hemispheres and the dorso-lumbar region of the cord. Cultures from this exudation showed in each the presence of Weichselbaum's diplococcus, and in the second case the pneumococcus also. The brain and cord from one case were also shown. He also described a case of posterior basic meningitis in a child, which, after an illness of eight weeks duration, ended in recovery. Retraction of the head was very marked in this case.—Dr. Cecil Williams recalled four cases of cerebro-spinal meningitis occurring during an epidemic of influenza in Wales in 1891.—Dr. H. Waldo thought hydrocephalus a valuable symptom, so that frequent measurements of the head should be made.—Dr. T. Fisher showed some sketches of cases of posterior basal meningitis, to show the retraction of the head. —The President thought it very difficult to differentiate between those due to middle-ear disease and simple basal meningitis. Would lumbar puncture assist?—Dr. Clarke replied that the fever in septic cases would be higher, and there would be rigors; the whole case would, in short, be more severe. He thought it probable that lumbar puncture would assist the diagnosis. His patient had no hydrocephalus.

Dr. Cecil Willliams read a paper on some points on the artificial feeding of infants. After referring to the difference between human and cow's milk, he passed to the value of the various ingredients and the manner in which cow's milk may be treated to render it suitable for young children. He referred to the methods of preparing humanised milk, and the use of whey and proprietary foods. He classified the latter, and mentioned their value from different points of view.

Bertram M. H. Rogers, *Reporter.*
J. Paul Bush, *Hon. Sec.*

